# Public Dental Dispensaries

**Published:** 1921-04

**Authors:** Julio Endelman


### PUBLIC DENTAL DISPENSARIES

JULIO ENDELMAN, D.D.S.

It is a matter of serious concern to those members of the dental profession who are interested in preventive dentistry, to realize the fact that comparatively little good will be accomplished without the moral and financial help of state and federal governments. The oral hygiene problem seems to be clearly divided into two phases, one of these having to do with the spreading of information concerning the importance of sound teeth and of the mouth as a whole, for its own sake, and as a factor in the conservation of human health, while the other involves the question of providing dental services to those unable to meet the average financial remuneration of private practitioners.

Important as is the education, along dental lines, of school children, it is of just as much importance that means be provided to take care of those children and adults in whom dental disease is already implanted, for the duel purpose of decreasing or abolishing a tremendous amount of avoidable pain and suffering and of increasing the physical and mental efficiency of thousands upon thousands of men and women throughout the nation. Lectures upon oral hygiene are increasing the demand for dental services; we are telling the people that the teeth must be taken care of, if they are to be free from those diseases which originate in infected root apices and investing tissues; we are telling them that the race is physically and mentally retrograding because of neglect of the mouth; and to all of this should we not add the instructions as to how, when and where these services are to be obtained at a cost within the limits of their financial resources? The oral hygiene campaign as carried out by means of lectures and demonstrations, is helping to mold public opinion as to the need of dental clinics under state supervision, where dental services may be obtained either free from all cost, or at fees in proportion to the means of the vast majority of those in need of dental attention. To merely announce the fact that dental disease is on the upward trend, that nine out of every ten children under fifteen years of age are dental cripples, that heart disease is yearly increasing, that the arthritides are incapacitating more and more people as time goes on, is to point out the menace without providing the means of averting it. It is a half-hearted, cold-blooded and inhuman proposition.

In those public institutions in which the inmates are supposed to have the advantage of dental services, it is the rule and not the exception that the number of dental operators is inadequate in proportion to the number of patients to be cared for, and the character of the services that should be rendered. Dental facilities seem to be provided in order to comply with certain regulations, and not upon that basis of appreciation of the needs of patients for dental treatment that would secure a sufficient number of competent operators, who would then possibly render thorough and not almost invariably services of a radical nature.

The efforts of a few enthusiasts in the field of prevention of dental disease have not received that whole-hearted approval of those who because of their official positions should be familiar with the social, economical and humanitarian significance of this great problem. The time has now arrived, when for the same reasons for which there are now in existence city, county and state hospitals, there should be organized alongside of them dental clinics for the care of those unable to defray the cost of dental treatment, as dispensed in private dental offices. The oral hygiene movement should contemplate not only the prevention of dental disease in the coming generations, but likewise the relief of conditions already at work in the mouths of four-fifths of our population. The inmates of public institutions represent but a small percentage of the number in dire need of dental attention. It is the great mass of toilers who are able, from the fruit of their work, to do little more than to supply their families and themselves with habitation and food, to say nothing of the many more sources of expense incident to the rearing of a family, that should be given the opportunity to receive dental treatment. These people either do without any dental services at all, or else receive a kind of so-called dental services, in the end more injurious to their future health and contentment than if their dental ills had been allowed to proceed undisturbed.

Is this not a matter that should receive proper consideration from federal and state departments of public health? Has not the matter been called to their attention heretofore in definite and explicit form? Aside from enterprise by private initiative, it would be interesting to determine what this state has accomplished in the prevention and treatment of dental disease, as well as the measures, if any, that are to be instituted in the near, nay nearest, future to correct a painfully neglected condition of affairs which is vitally affecting and undermining the health of a vast majority of the men, women and children of this commonwealth.

The following data from a report on the effects of dental treatment on national health and physique compiled by Dr.

Herbert L. Wheeler * of New York, may serve a good purpose in pointing out to our various city, county and state authorities, the economical significance of services rendered under their auspices:

New York City through the Bureau of Child Hygiene of the Department of Health, has demonstrated a reduction of twenty-five per cent. of absences due to oral troubles in those schools in which a dental service has been established. Furthermore, in New York City, 140,000 children are repeaters. Around fifty per cent. of this number are held back on account of oral conditions. It costs in New York on an average of $49 (in 1914) per pupil annually to carry a child a year in the public schools, or a total cost for the fifty per cent. retardations due to oral conditions, of around three and one-half million dollars per year. Dental attention which would have prevented these retardations or necessity of repeating the year, could have been given at a total cost of between four hundred and fifty and five hundred thousand dollars. In other words, by giving dental treatment to the school children of the city, besides rendering them physically and mentally able, a saving of around three millions would have been realized. All of this applies with equal force to any and all cities of the country.

If not for humanitarian reasons or for reasons encompassing racial improvement, certainly for economic reasons should dental clinics be established throughout every state of the union. It will be a paying investment to the state in actual dollars and cents, and above all in human happiness and efficiency, in which we are or should be mostly concerned.

---

* "Report on Effects of Dental Treatment on National Health and Physique, Transactions of the Sixth International Dental Congress, London, 1914, page 575."

